# Supplementary material for: Polarization manipulation of electromagnetic interference shielding effectiveness utilizing graphene film-based metamaterials
Source: Nat Commun. 2025 Dec 11;17:682. doi: 10.1038/s41467-025-67335-x (PMC12820276; doi:10.1038/s41467-025-67335-x)
Supplement: Supplementary file 1 — Supplementary Information [file 41467_2025_67335_MOESM1_ESM.pdf]

Supplementary Information

Polarization Manipulation of Electromagnetic Interference Shielding Effectiveness Utilizing Graphene Film-Based Metamaterials

Zhe Wang[1,2], Haoran Zu[2,3,*], Zhi Luo[2], Yu Zhou[2], Yiping Ren[2], Huazhang Zhang[2], Zixin Zhang[2], Wei Qian[2], Huaqiang Fu[2], Lun Li[2], Hao Feng[1], Pengfei Chen[2], Long Zhang[1], Hao Yuan[1], Junkang Xia[1], Xin Zhao[2], Shuxin Li[1,*], Daping He[2,*]

[1] *State Key Laboratory of Advanced Technology for Materials Synthesis and Processing, Wuhan University of Technology, Wuhan 430070, P. R. China*

[2] *Hubei Engineering Research Center of Radio Frequency Microwave Technology and Application, Wuhan University of Technology, Wuhan 430070, P. R. China*

[3] *School of Information Engineering, Wuhan University of Technology, Wuhan 430070, P. R. China*

** Corresponding author E-mail: zuhr@whut.edu.cn (H. Zu), lishuxin@whut.edu.cn (S. Li), hedaping@whut.edu.cn (D. He)*

This document includes:

- Supplementary Figure 1 to Figure 16 and Table 1 to Table 2
- Supplementary Note 1. Experimental details
- Supplementary Note 2. The principle of high-polarization sensitivity of GAFM
- Supplementary Note 3. The EMI SE measurement procedures
- Supplementary References

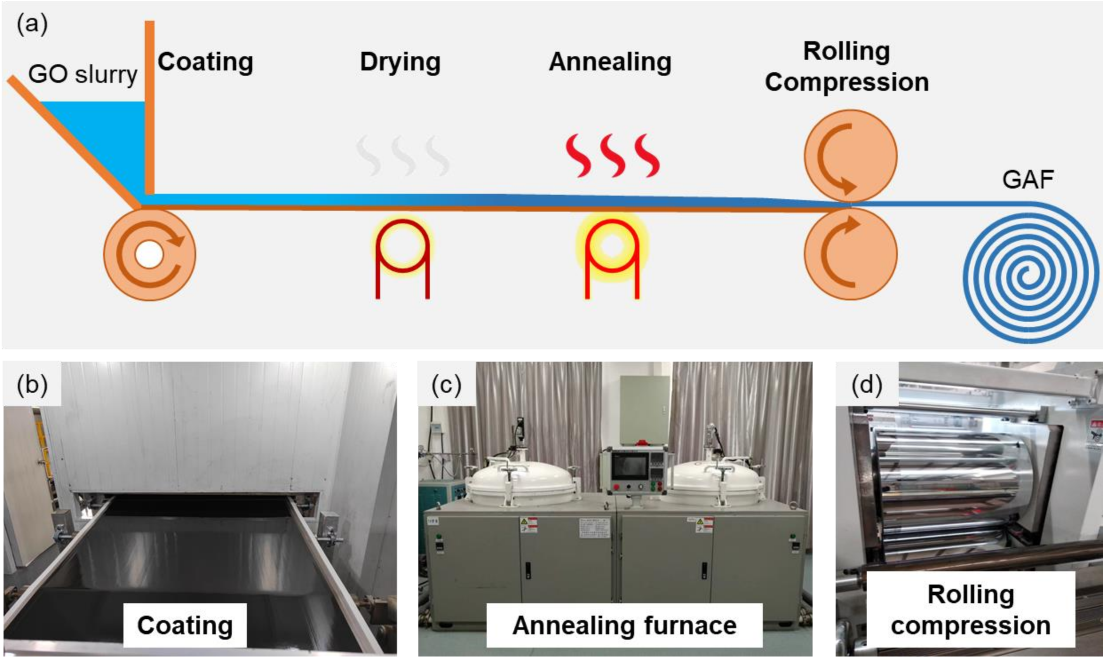

Supplementary Fig. 1. The fabrication process of graphene assembled film (GAF) based on graphene oxide (GO). (a) Schematic diagram of GAF preparation process. Images of (b) coating, (c) annealing furnace, and (d) rolling compression.

As shown in **Supplementary Fig. 1**, the GAF is synthesized through a process involving high-temperature annealing followed by rolling compression treatment applied to graphene oxide (GO) assembly films.

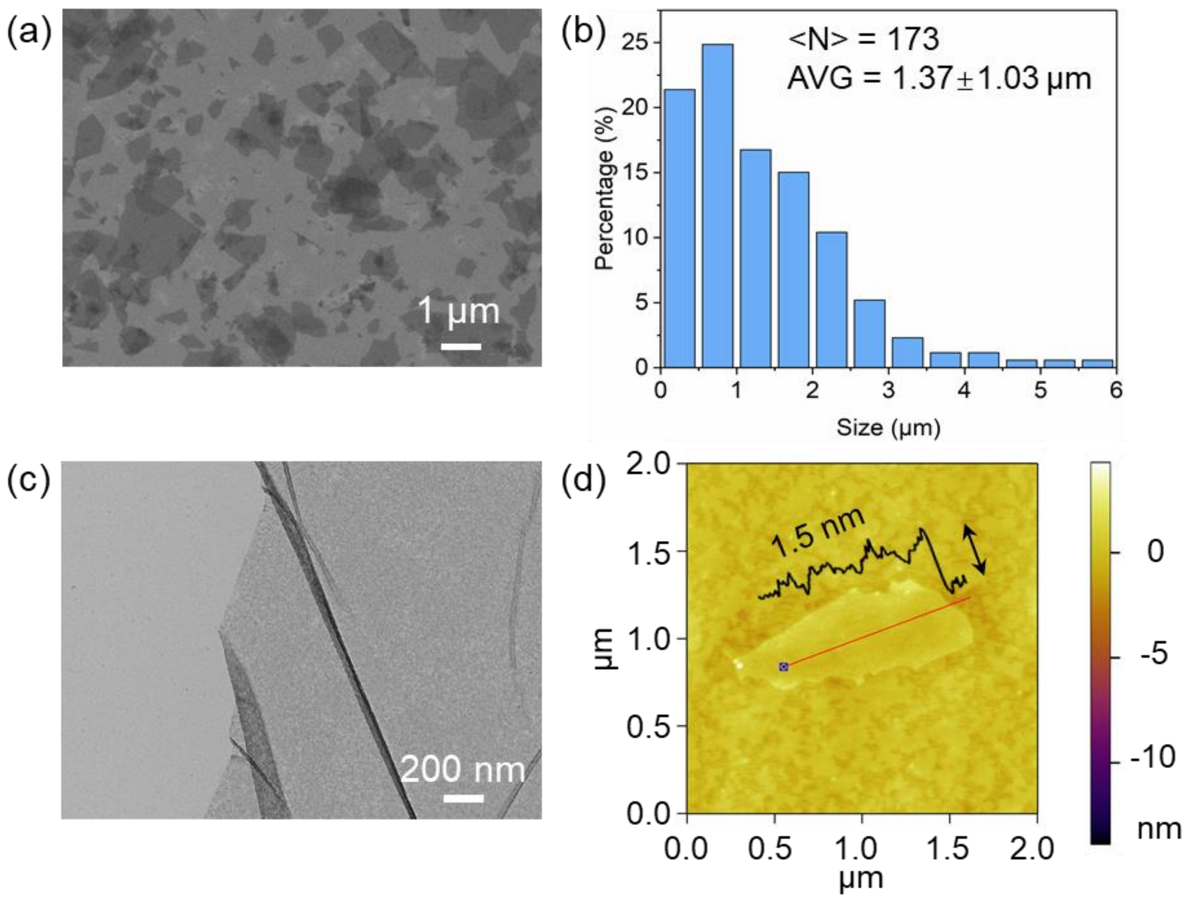

Supplementary Fig. 2. Characterization of GO sheets. (a) SEM micrographs and (b) corresponding size distribution of GO sheets, (c) TEM image and (d) AFM ichnography of GO sheets. Data are representative of five independent experiments with similar results.

As depicted in **Supplementary Fig. 2**, the GO sheets exhibit an average lateral size of 1.37 ± 1.03 μm. The microstructure of GO sheets is characterized using transmission electron microscopy (TEM) and atomic force microscopy (AFM).

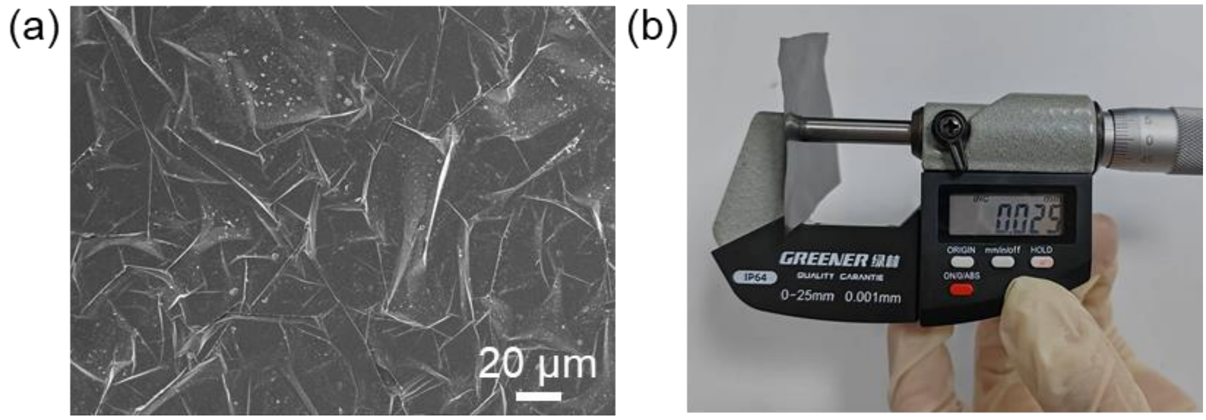

Supplementary Fig. 3. Morphological characterization of GAF. (a) Surface scanning electron microscope (SEM) image, and (b) image of GAF with 25 μm thickness. Data are representative of five independent experiments with similar results.

Supplementary Fig. 3a depicts the top-view scanning electron microscopy (SEM) image of the GAF, revealing a uniform distribution of micro-folds across the GAF surface. This distribution is attributed to the synergistic effects of graphene nanosheet stacking, the conversion of oxygen-containing functional groups into gases (i.e. CO, CO_2, H_2O), and the subsequent formation of micro-cavities that evolve into micro-folds. Additionally, **Supplementary Fig. 3b** illustrates GAF with a measured thickness of 25 μm.

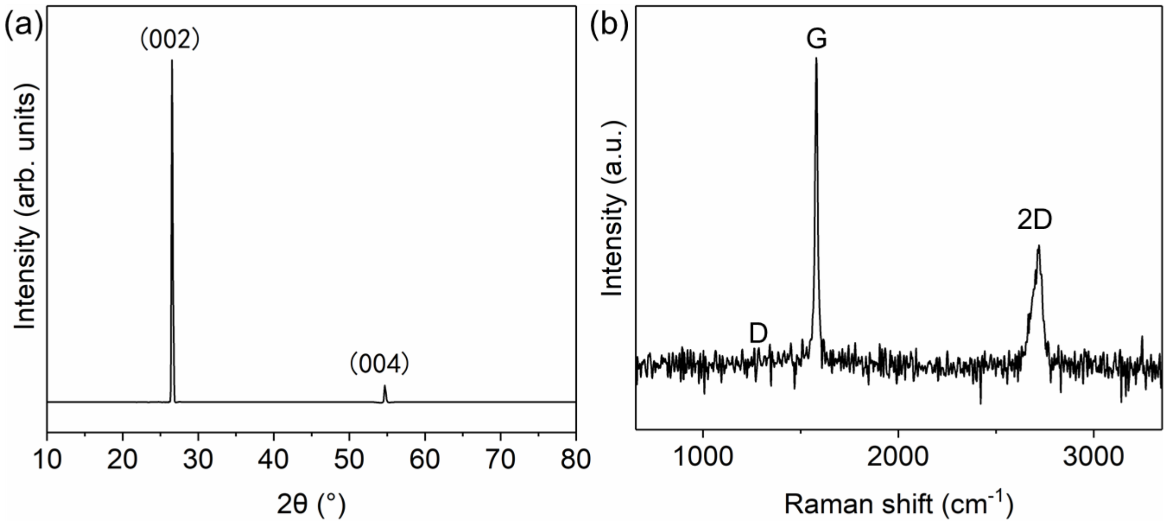

Supplementary Fig. 4. Characterization of GAF. (a) X-ray diffraction (XRD) pattern and (b) Raman spectroscopy of the GAF.

The X-ray diffraction (XRD) pattern of the GAF reveals a distinct diffraction peak at 26.5°, indicative of a *d*-spacing of 0.34 nm corresponding to the (002) plane, affirming its highly graphitized structure (**Supplementary Fig. 4a**). Furthermore, the presence of the (004) peak further confirms the ordered arrangement of the graphene laminates. Moreover, as shown in **Supplementary Fig. 4b**, the Raman spectroscopy analysis of the GAF demonstrates prominent peaks of G band (1580 cm^{-1}) and 2D band (2719 cm^{-1}), without significant observation of the D band. This absence suggests the effective elimination of lattice defects and the formation of characteristic sp^2 hybridized lattices within the graphene structure.

.

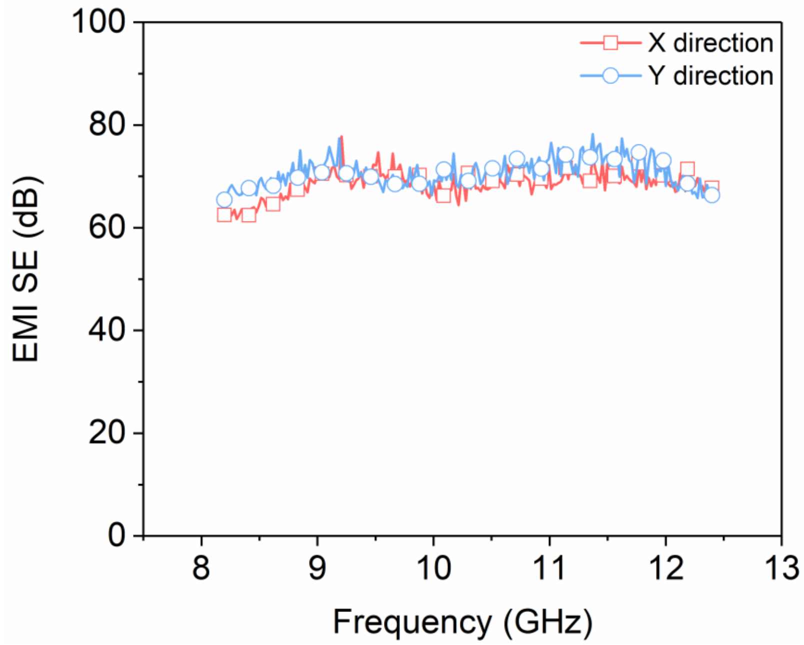

Supplementary Fig. 5. The electromagnetic interference shielding effectiveness (EMI SE) of GAF in X and Y direction.

The consistent electromagnetic interference shielding effectiveness (EMI SE) of the GAF in both the X and Y directions confirms its isotropic nature within the plane (**Supplementary Fig. 5**).

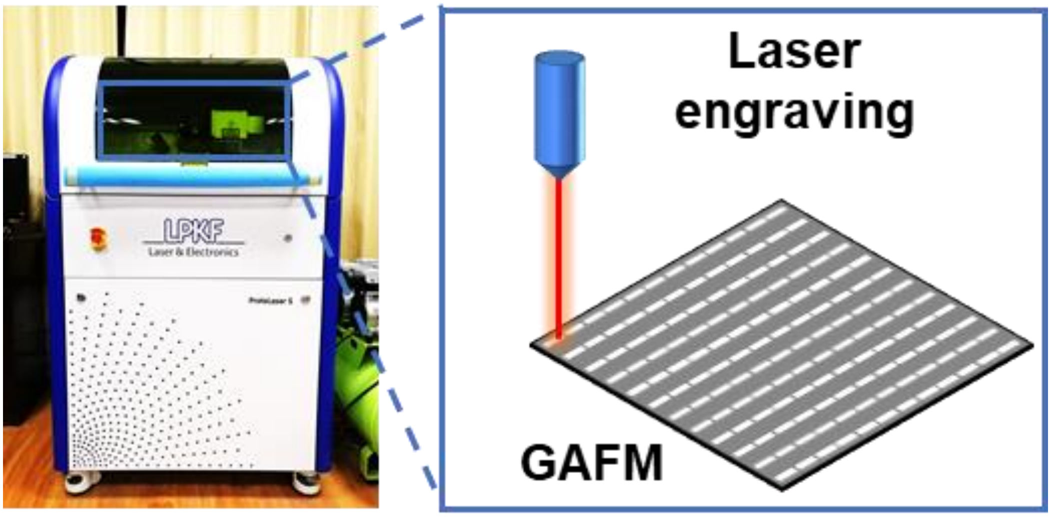

Supplementary Fig. 6. The graphene assembled film metamaterials (GAFM) was prepared from GAF by laser engraving method.

As shown in **Supplementary Fig. 6**, the geometric dimensions of the pre-designed graphene assembled film metamaterials (GAFM) were fed into the LPKF CircuitPro PL 2.0 computational simulation software. Subsequently, using a laser engraving machine (LPKF Laser & Electronics ProtoLaser S), GAFM was manufactured in one step according to the calculated laser path.

Supplementary Table 1. Comparison of EMI shielding efficiency for "On" state and "Off" state, switching efficiency using different strategies, and other performances reported in the references at X band (8.2 - 12.4 GHz).

**No.**	**Ref.**	**Materials**	**EMI shielding efficiency for "ON" state (η_{ON}, %)**	**EMI shielding efficiency for "OFF" state (η_{OFF}, %)**	**Switching efficiency (η_{OFF}-η_{ON}, %)**	**Full polariz-ation shielding**	**Enhan-ced polariz-ation sensiti-vity**	**Type**
1	S1	Wood-derived carbon/XC-72 NP aerogel	29.2054216 (Y-polarization)	99.7181617 (Y-polarization)	70.5127401	NO	NO	Shape change (compress-ion)
2	S2	TPI-MXene/carbon foam	96.8377223 (Y-polarization)	99.6837722 (Y-polarization)	2.8460499	NO	NO	Shape change (compress-ion)
3	S3	EVA@PPy@Ag foam	76.1768053 (Y-polarization)	99.9999308 (Y-polarization)	23.8231255	NO	NO	Shape change (compress-ion)
4	S4	MF@MXene/Ag NW sponges/PEG	94.2456006 (Y-polarization)	99.9108749 (Y-polarization)	5.6652743	NO	NO	Shape change (compress-ion)

No.	Ref.	Materials	EMI shielding efficiency for "ON" state (η_{ON}, %)	EMI shielding efficiency for "OFF" state (η_{OFF}, %)	Switching efficiency (η_{OFF}-η_{ON}, %)	Full polariz-ation shielding	Enhan-ced polariz-ation sensiti-vity	Type
5	S5	WF/CNTs foam	95.9261972 (Y-polarization)	99.9902276 (Y-polarization)	4.0640304	NO	NO	Shape change (compress-ion)
6	S6	PU/CNTs/TPI	99.5831306 (Y-polarization)	99.9668869 (Y-polarization)	0.3837563	NO	NO	Shape change (compress-ion)
7	S7	MF@Ag/PDA/CNTs/ waterborne PU	99.6980048 (Y-polarization)	99.9754529 (Y-polarization)	0.2774481	NO	NO	Shape change (compress-ion)
8	S8	Identical patterned anisotropic magnetic liquid metal/PDMS	87.4107459 (Y-polarization)	99.9936904 (Y-polarization)	12.5829445	NO	NO	Shape change (compress-ion)
9	S9	PU/RGO foam-5%	96.8377223 (Y-polarization)	99.4988128 (Y-polarization)	2.6610904	NO	NO	Shape change (compress-ion)

No.	Ref.	Materials	EMI shielding efficiency for "ON" state (η_{ON}, %)	EMI shielding efficiency for "OFF" state (η_{OFF}, %)	Switching efficiency (η_{OFF}-η_{ON}, %)	Full polariz-ation shielding	Enhan-ced polariz-ation sensiti-vity	Type
10	S9	PU/RGO foam-10%	99.3690427 (Y-polarization)	99.9936904 (Y-polarization)	0.6246478	NO	NO	Shape change (compress-ion)
11	S10	Pre-linked Ni chains elastomer	36.9042656 (Y-polarization)	99.3690427 (Y-polarization)	62.4647771	NO	NO	Shape change (stretching)
12	S11	CNT@liquid metal/ polyacrylamide/gelatin	49.8812766 (Y-polarization)	99.9900000 (Y-polarization)	50.1087234	NO	NO	Shape change (stretching)
13	S12	Fe/liquid metal/PDMS	99.1290364 (Y-polarization)	99.9999991 (Y-polarization)	0.8709627	NO	NO	Shape change (stretching)
14	S13	3D liquid metal network	99.9645187 (Y-polarization)	99.9999998 (Y-polarization)	0.0354811	NO	NO	Shape change (stretching)
15	S14	Liquid metal elastomer (BiInSn)	20.5671765 (Y-polarization)	92.0567177 (Y-polarization)	71.4895411	NO	NO	Shape change (stretching)
16	S14	Liquid metal elastomer (Ga)	92.0567177 (Y-polarization)	99.8004738 (Y-polarization)	7.7437561	NO	NO	Shape change (stretching)

No.	Ref.	Materials	EMI shielding efficiency for "ON" state (η_{ON}, %)	EMI shielding efficiency for "OFF" state (η_{OFF}, %)	Switching efficiency (η_{OFF}-η_{ON}, %)	Full polarization shielding	Enhanced polarization sensitivity	Type
17	S14	Liquid metal foamed elastomer (Ga)	99.9999000 (Y-polarization)	99.9999997 (Y-polarization)	0.0000997	NO	NO	Shape change (stretching)
18	S15	Ag NPs/SEBS	99.8415107 (Y-polarization)	99.9996838 (Y-polarization)	0.1581731	NO	NO	Shape change (stretching)
19	S16	CNT/TPU	94.3765867 (Y-polarization)	99.9653263 (Y-polarization)	5.5887396	NO	NO	Shape change (stretching)
20	S17	Liquid metal GaIn24.5/Ni	99.9999000 (Y-polarization)	99.9999900 (Y-polarization)	0.0000900	NO	NO	Shape change (stretching)
21	S18	PEDOT:PSS/ waterborne PU	99.9996019 (Y-polarization)	99.9998415 (Y-polarization)	0.0002396	NO	NO	Shape change (stretching)
22	S19	VO_2/CNF	98.6510371 (Y-polarization)	99.9994752 (Y-polarization)	1.3484381	NO	NO	Temperature change
23	S20	VO_2/EPM foam	5.59391237 (Y-polarization)	52.6848741 (Y-polarization)	47.0909617	NO	NO	Temperature change

No.	Ref.	Materials	EMI shielding efficiency for "ON" state (η_{ON}, %)	EMI shielding efficiency for "OFF" state (η_{OFF}, %)	Switching efficiency (η_{OFF}-η_{ON}, %)	Full polariz-ation shielding	Enhan-ced polariz-ation sensiti-vity	Type
24	S21	VO_2/PVDF-HFP	80.0473769 (Y-polarization)	99.9993393 (Y-polarization)	19.9519625	NO	NO	Temperature change
25	S22	$Ti_3C_2T_x$-WVO_2	99.8711750 (Y-polarization)	99.9947519 (Y-polarization)	0.1235769	NO	NO	Temperature change
26	S23	RGO/VO_2-300℃	60.1892829 (Y-polarization)	92.0567177 (Y-polarization)	31.8674347	NO	NO	Temperature change
27	S23	RGO/VO_2	84.1510681 (Y-polarization)	99.9996019 (Y-polarization)	15.8485338	NO	NO	Temperature change
28	S24	Graphene/PDMS	99.9910875 (Y-polarization)	99.9205672 (Y-polarization)	0.0705203	NO	NO	Temperature change
29	S25	Core-shell structural PNIPAM@p-PDA biomicrospheres	99.9601893 (Y-polarization)	99.99999997 (Y-polarization)	0.0398107	NO	NO	Humidity change
30	S26	Pyrolytic graphite-wet RGO/CNTs/PP non-woven spacer-pyrolytic graphite	97.4881136 (Y-polarization)	99.9841511 (Y-polarization)	2.4960375	NO	NO	Humidity change

No.	Ref.	Materials	EMI shielding efficiency for "ON" state (η_{ON}, %)	EMI shielding efficiency for "OFF" state (η_{OFF}, %)	Switching efficiency (η_{OFF}-η_{ON}, %)	Full polariz-ation shielding	Enhan-ced polariz-ation sensiti-vity	Type
31	S27	MXene film ($Ti_3C_2T_x$ electrode in 1 M H_2SO_4)/PET	99.9205672 (Y-polarization)	99.9521370 (Y-polarization)	0.0315698	NO	NO	Electroch-emical potential change
32	S27	MXene film (V_2CT_x electrode in 1 M H_2SO_4)/PET	96.9800483 (Y-polarization)	99.2414224 (Y-polarization)	2.2613741	NO	NO	Electroch-emical potential change
33	S28	MXene/CNF aerogels	99.9800474 (X-polarization)	99.9999499 (Y-polarization)	0.0199025	YES	NO	Rotation angle change
34	S29	MXene@wood	99.8180299 (X-polarization)	99.9998262 (Y-polarization)	0.1817963	YES	NO	Rotation angle change
35	S30	Polyvinyl butyral/Ni-graphene/short-cut CF films	99.0000000 (X-polarization)	99.9205672 (Y-polarization)	0.9205672	YES	NO	Rotation angle change
36	S31	CF reinforced polymer	90.0000000 (X-polarization)	99.9987411 (Y-polarization)	9.9987411	YES	NO	Rotation angle change
37	S32	CF hybrid fabrics	43.7658675 (X-polarization)	80.0473769 (Y-polarization)	36.2815094	NO	NO	Rotation angle change

No.	Ref.	Materials	EMI shielding efficiency for "ON" state (η_{ON}, %)	EMI shielding efficiency for "OFF" state (η_{OFF}, %)	Switching efficiency (η_{OFF}-η_{ON}, %)	Full polarization shielding	Enhanced polarization sensitivity	Type
38	S33	RGO@directional porous carbon	99.6764063 (X-polarization)	99.9597283 (Y-polarization)	0.2833220	YES	NO	Rotation angle change
39	S34	CNT/NFC	99.9553316 (X-polarization)	99.9999937 (Y-polarization)	0.0446620	YES	NO	Rotation angle change
40	S35	Co based amorphous wires	12.9036410 (X-polarization)	99.6611558 (Y-polarization)	86.7575148	NO	NO	Rotation angle change
41	S36	MXene@PDA	36.9042656 (X-polarization)	98.7410746 (Y-polarization)	61.8368090	NO	NO	Rotation angle change
42	S37	Balsa tangential-section carbonized wood	99.9000000 (X-polarization)	99.9999990 (Y-polarization)	0.0999990	YES	NO	Rotation angle change
43	S38	Ordered RGO fiber film	68.3772234 (X-polarization)	99.9205672 (Y-polarization)	31.5433438	NO	NO	Rotation angle change
44	S39	CNT-based spacer fabric with CVD	99.9498813 (X-polarization)	99.9999998 (Y-polarization)	0.0501185	YES	NO	Rotation angle change

No.	Ref.	Materials	EMI shielding efficiency for “ON” state (η_{ON}, %)	EMI shielding efficiency for “OFF” state (η_{OFF}, %)	Switching efficiency (η_{OFF}-η_{ON}, %)	Full polarization shielding	Enhanced polarization sensitivity	Type
45	This work	GAFM	3.1722144 (X-polarization)	99.7852170 (Y-polarization)	96.6130026	YES (+⊥CF)	YES (+//CF)	Rotation angle change

Note: nanoparticles (NP), trans-1,4-polyisoprene (TPI), ethylene–vinyl acetate copolymer (EVA), polypyrrole (PPy), silver (Ag), melamine foam (MF), nanowire (NW), poly(ethylene glycol) (PEG), wheat flour (WF), carbon nanotube (CNT), polyurethane (PU), poly-dopamine (PDA), polydimethylsiloxane (PDMS), reduced graphene oxide (RGO), 3 dimension (3D), styrene-(ethylenebutylene)-styrene (SEBS), thermoplastic polyurethane (TPU), cellulose nanofibrils (CNF), poly(3,4-ethylenedioxythiophene)/poly(styrenesulfonate) (PEDOT:PSS), carbon fiber (CF), vanadium dioxide (VO_2), expanded polymer microsphere (EPM), poly (vinylidenefluoride-co-hexafluoropropylene (PVDF-HFP), poly(N-isopropylacrylamide)@ porous polydopamine (PNIPAM@p-PDA), polyprolene (PP), poly(ethylene terephthalate) (PET), nano-brillated cellulose (NFC), chemical vapor deposition (CVD). Full polarization shielding refers to the EMI shielding efficiency being higher than 90% in both the X and Y polarization.

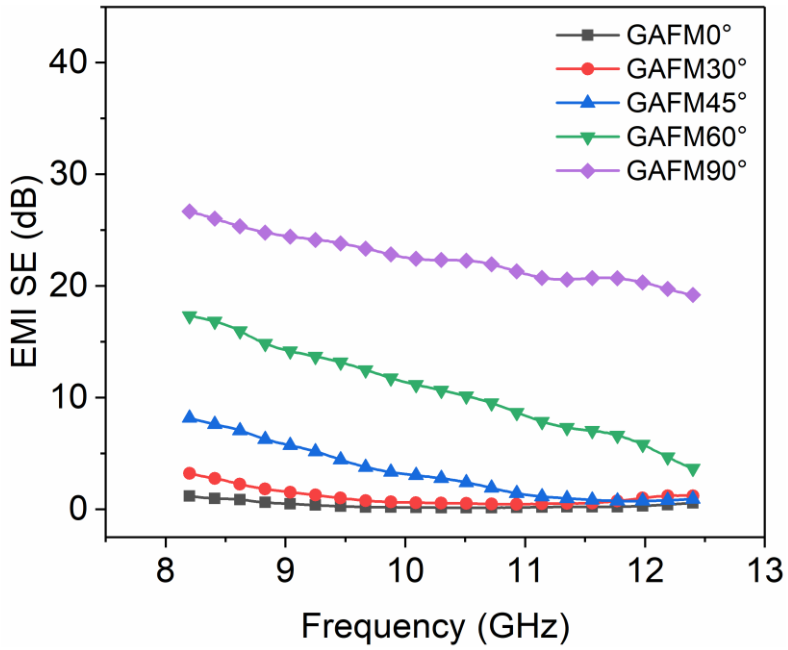

Supplementary Fig. 7. The EMI SE of GAFM when the rotation angle is from 0° to 90°.

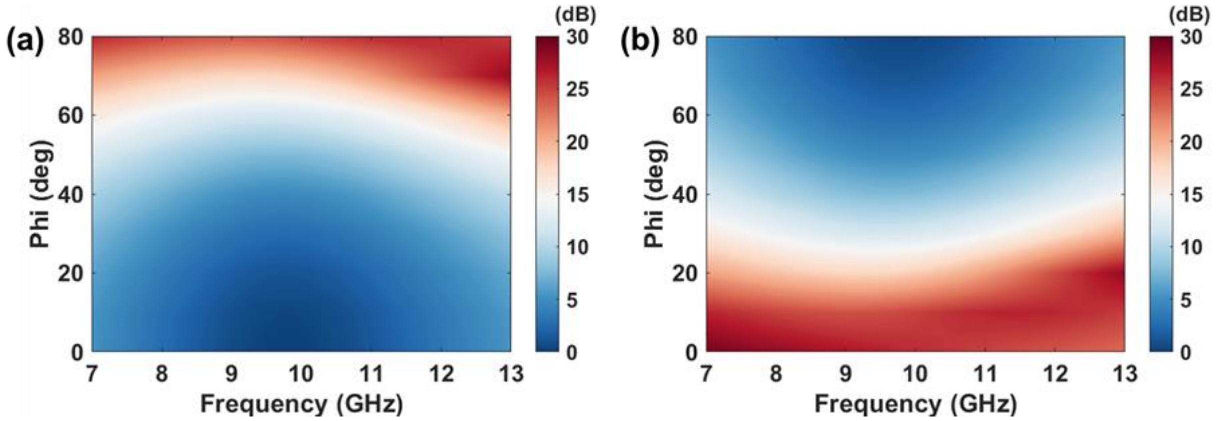

Supplementary Fig. 8. The EMI SE of GAFM in X-band at (a) Y polarization and (b) X polarizations varies with the change of polarization angle.

(a)

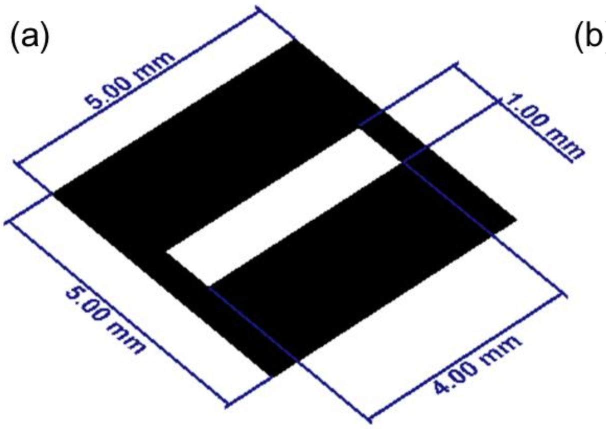

(b)

Geometric parameters	Size (mm)
l	5
w	5
t	0.025
sl	1
sw	4

(c)

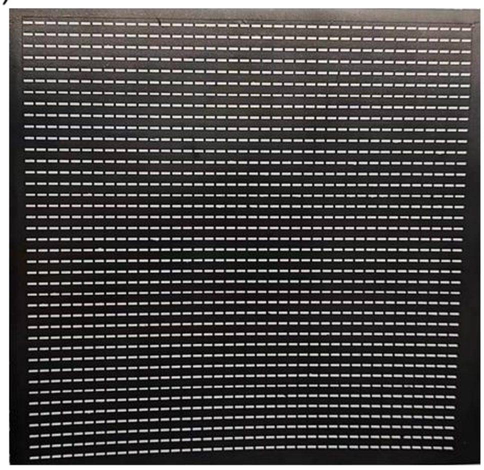

(d)

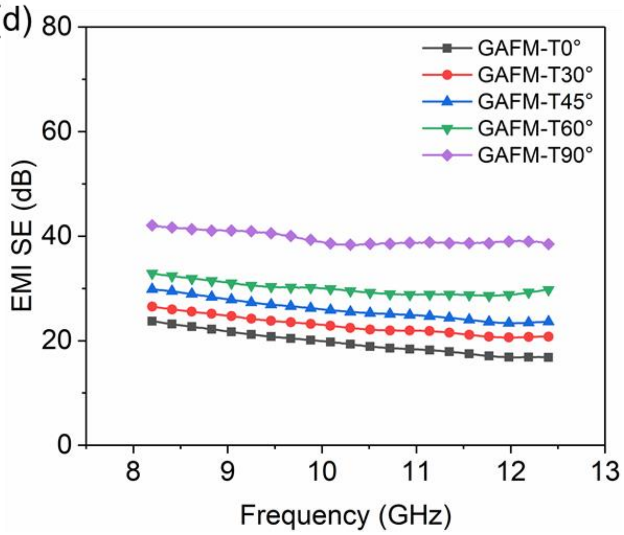

Supplementary Fig. 9. The geometric parameters and EMI SE of typical GAFM (GAFM-T). (a,b) Geometric parameters of GAFM-T unit cell, with l = 5 mm, w = 5 mm, sl = 1 mm, sw = 4 mm, t = 0.025 mm. (c) Digital image of GAFM-T. (d) Measured EMI SE of GAFM-T at different rotation angles.

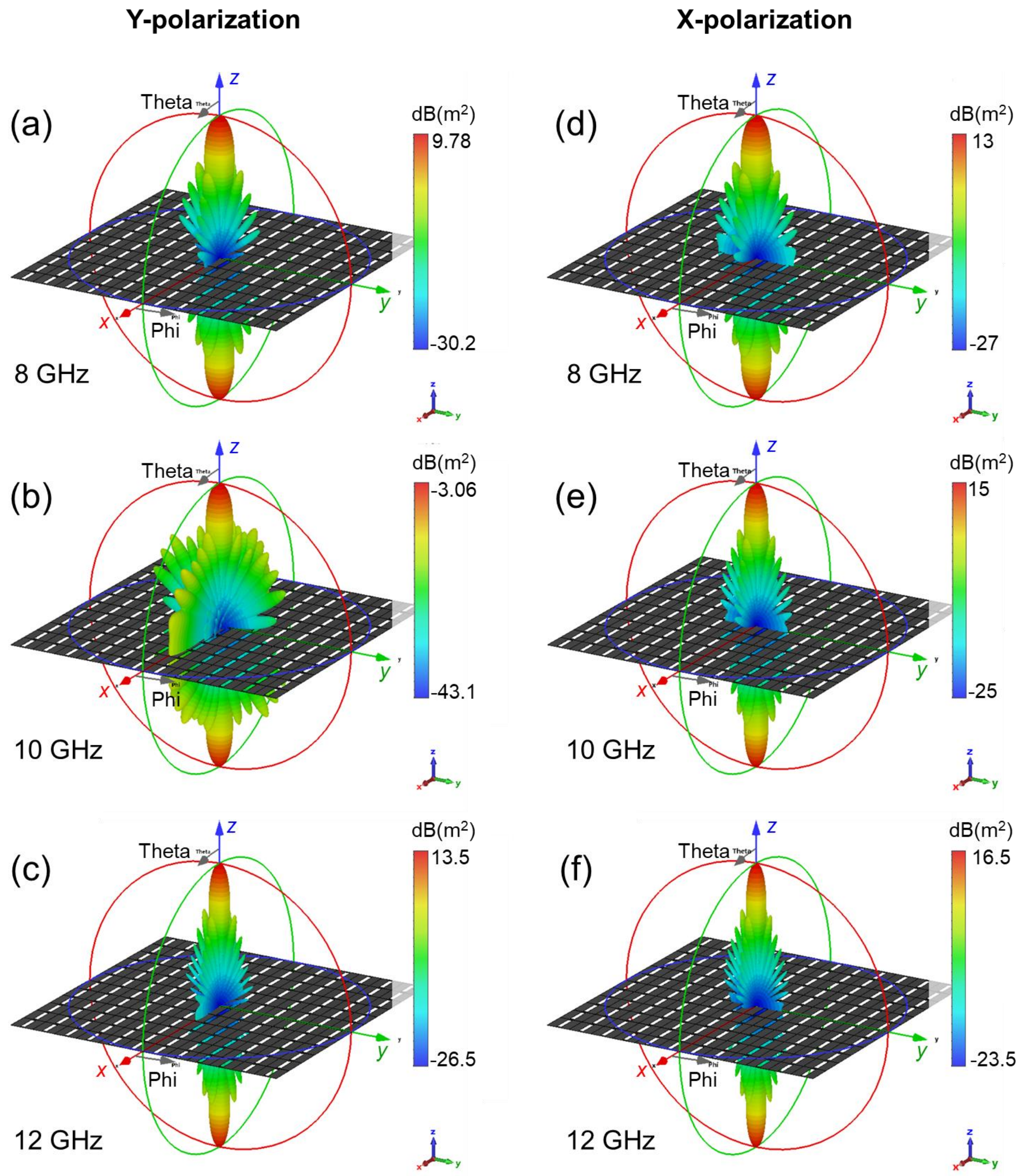

Supplementary Fig. 10. 3D far-field radiation patterns of the GAFM at 8 GHz, 10 GHz, and 12 GHz for plane waves with (a-c) Y-polarization and (d-f) X-polarization.

To effectively analyze the non-specular reflection modes of the GAFM, as illustrated in **Supplementary Fig. 10**, we conducted simulations using CST to examine the far-field radiation patterns of a 10 × 10 GAFM array. Plane electromagnetic waves polarized in the Y and X directions were used as excitation sources. Under conditions of normal incidence, the primary

reflected beam of the GAFM array aligns perpendicularly with the incident direction, thereby demonstrating the absence of non-specular reflection phenomena. Additionally, at 10 GHz, the intensity of the reflected beam for Y-polarized waves is significantly lower than at 8 GHz and 12 GHz, while X-polarized waves consistently exhibit higher amplitude reflections. These results align with individual element simulations.

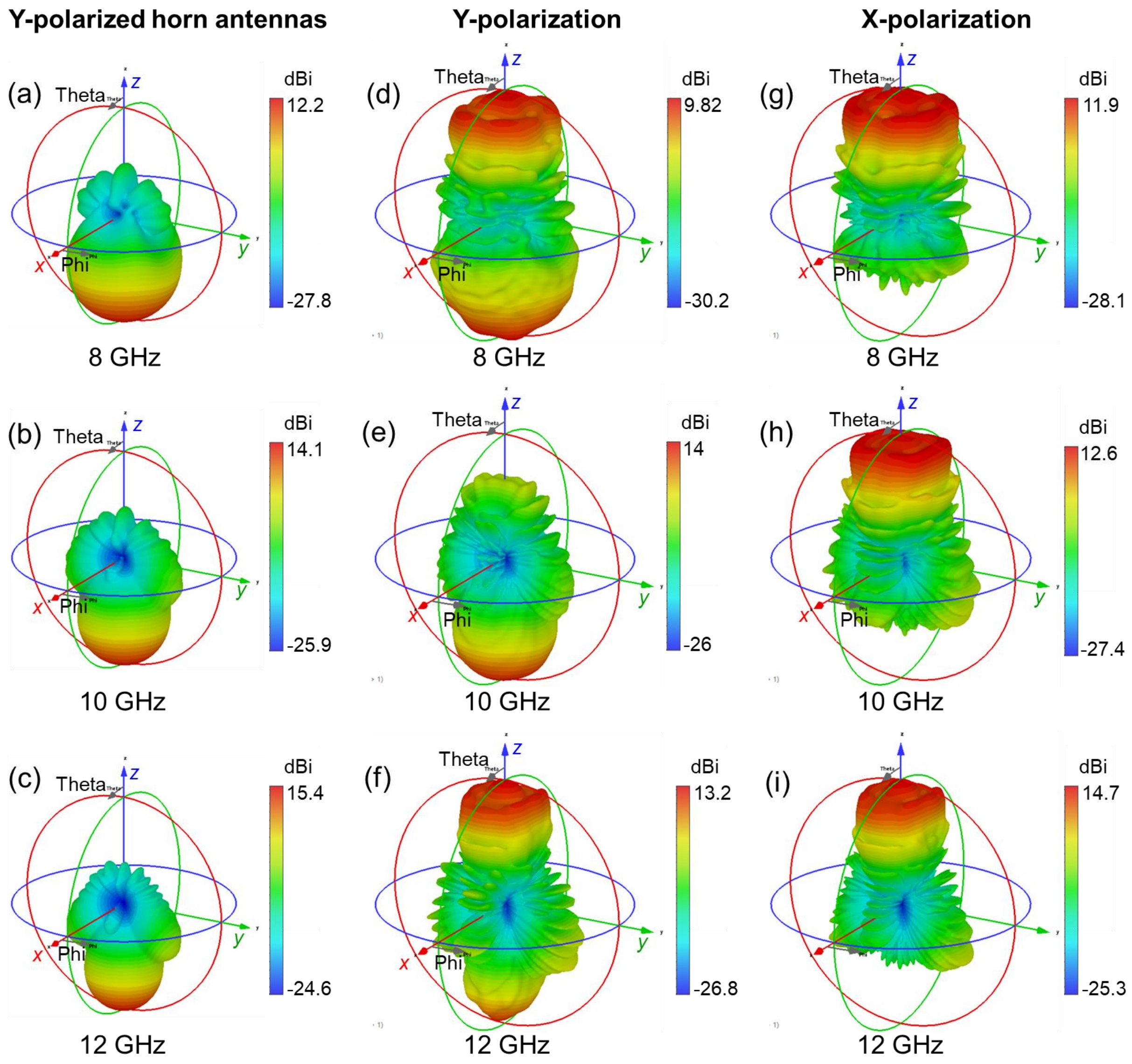

Supplementary Fig. 11. 3D far-field radiation patterns of Y-polarized horn antennas at (a) 8 GHz, (b) 10 GHz, and (c) 12 GHz. 3D far-field radiation patterns of the GAFM employing horn antennas as excitation sources at (d) 8 GHz, (e) 10 GHz, and (f) 12 GHz with Y-polarization; and the corresponding patterns at (g) 8 GHz, (h) 10 GHz, and (i) 12 GHz with X-polarization.

For further analysis, a horn antenna was used as the feed source in simulations. **Supplementary Fig. 11a-11c** show the far-field radiation patterns of the horn antenna, and **Supplementary Fig. 11d-11i** depict the far-field beams of the GAFM array with the horn. The results obtained from horn feeds with different polarization orientations are consistent with those derived from plane wave simulation, confirming the reliability of our approach.

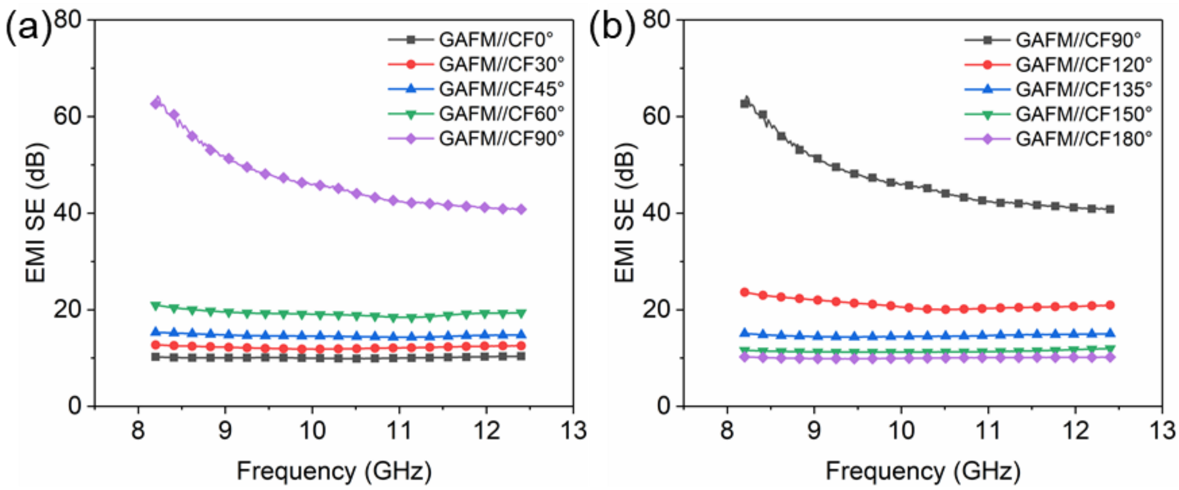

Supplementary Fig. 12. The EMI SE polarization manipulation at X band (8.2 ~ 12.4 GHz) of GAFM // CF. (a) The EMI SE polarization manipulation of GAFM // CF when the rotation angle changes from 0° to 90°. (b) The EMI SE polarization manipulation of GAFM // CF when the rotation angle changes from 90° to 180°.

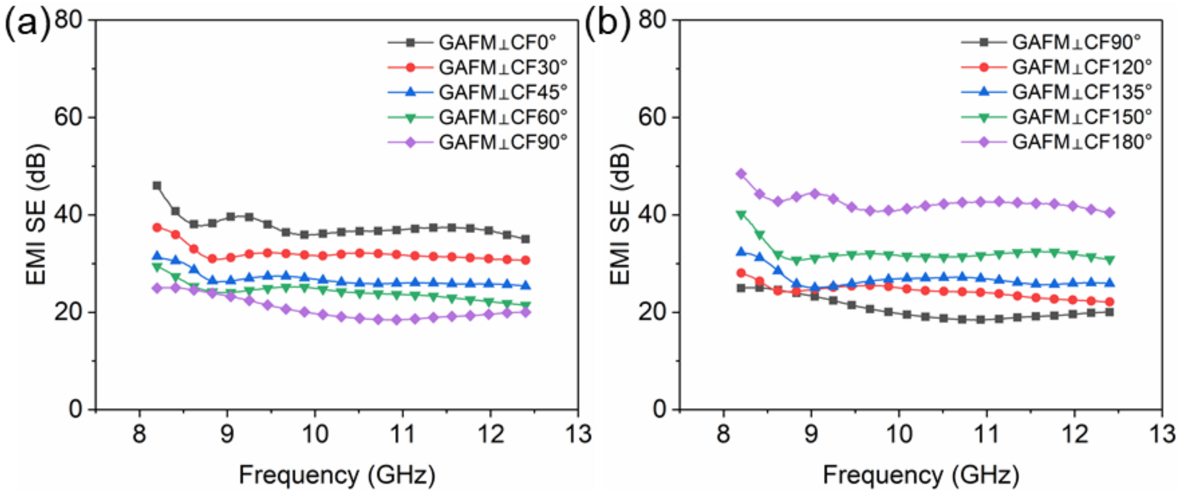

Supplementary Fig. 13. The EMI SE polarization manipulation at X band (8.2 ~ 12.4 GHz) of GAFM⊥CF. (a) The EMI SE polarization manipulation of GAFM⊥CF when the rotation angle changes from 0° to 90°. (b) The EMI SE polarization manipulation of GAFM⊥CF when the rotation angle changes from 90° to 180°.

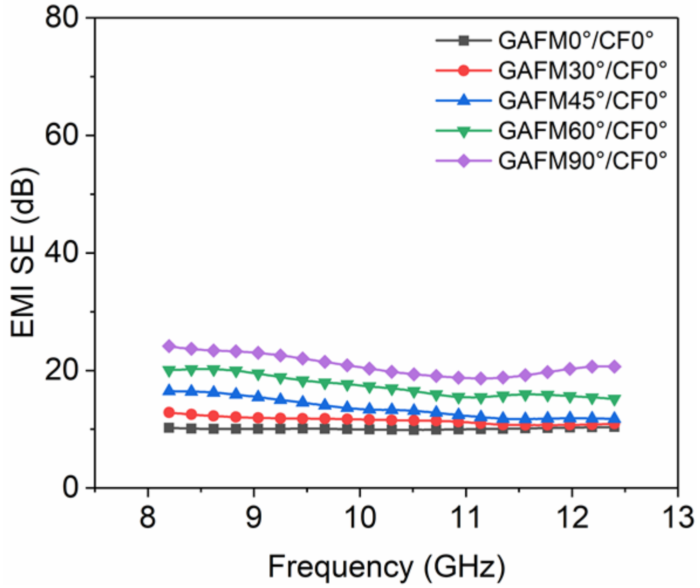

Supplementary Fig. 14. The EMI SE polarization manipulation at X band (8.2~12.4 dB) of the composite structure of GAFM and carbon fiber (GAFM/CF) in GAFM-rotated/CF-fixed state when the rotation angle changes from 0° to 90°.

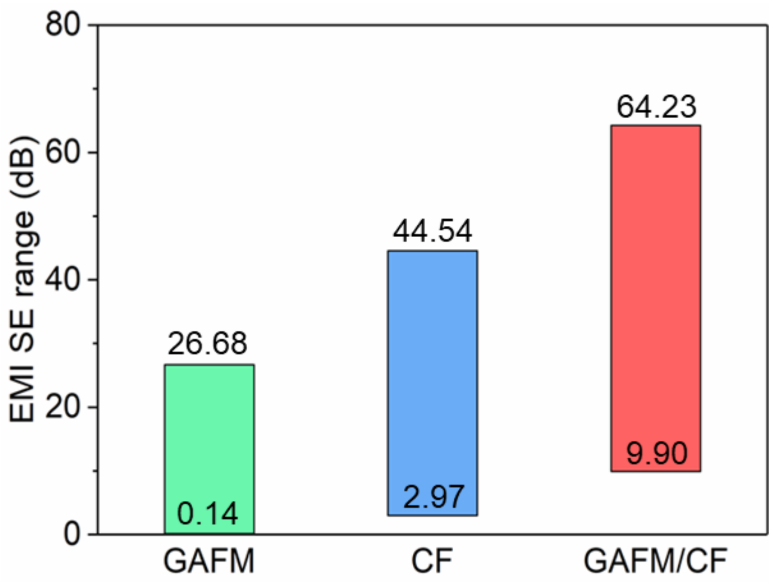

Supplementary Fig. 15. The EMI SE range of GAFM, carbon fiber (CF) and GAFM/CF.

Supplementary Table 2. Comparison of the EMI SE polarization manipulation difference (ΔEMI SE) and ΔEMI SE per unit thickness (ΔEMI SE/t) using different strategies (shape change, temperature change, humidity change, electrochemical potential change, and rotation angle change) reported in the references at X band (8.2 - 12.4 GHz).

No.	Type	Materials	ΔEMI SE (dB)	ΔEMI SE/t (dB/mm)	References
1	Shape change (compression)	Wood-derived carbon/XC-72 NP aerogel	24.00	0.34	S1
2	Shape change (compression)	TPI-MXene/carbon foam	10.00	1.00	S2
3	Shape change (compression)	EVA@PPy@Ag foam	45.00	22.50	S3
4	Shape change (compression)	MF@MXene/Ag NW sponges/PEG	18.10	9.05	S4
5	Shape change (compression)	WF/CNTs composite foam	26.20	5.24	S5
6	Shape change (compression)	PU/CNTs/TPI	11.00	1.10	S6
7	Shape change (compression)	MF@Ag/PDA/CNTs/ waterborne PU	9.90	15.00	S7
8	Shape change (compression)	Identical patterned anisotropic magnetic liquid metal/PDMS	33.00	21.88	S8
9	Shape change (compression)	PU/RGO foam-5%	8.00	0.13	S9

No.	Type	Materials	ΔEMI SE (dB)	ΔEMI SE/t (dB/mm)	References
10	Shape change (compression)	PU/RGO foam-10%	20.00	0.33	S9
11	Shape change (stretching)	Pre-linked Ni chains elastomer	20.00	66.67	S10
12	Shape change (stretching)	CNT@liquid metal/PAM/gelatin	37.00	9.25	S11
13	Shape change (stretching)	Fe/liquid metal/PDMS	40.10	50.13	S12
14	Shape change (stretching)	3D liquid metal network	51.70	32.31	S13
15	Shape change (stretching)	Liquid metal elastomer composite (BiInSn)	10.00	2.78	S14
16	Shape change (stretching)	Liquid metal elastomer composite (Ga)	16.00	4.44	S14
17	Shape change (stretching)	Liquid metal foamed elastomer composite (Ga)	25.00	6.94	S14
18	Shape change (stretching)	Ag NPs/SEBS	27.00	10.38	S15
19	Shape change (stretching)	CNT/TPU	22.10	11.05	S16
20	Shape change (stretching)	Liquid metal GaIn24.5/Ni	10.00	200.00	S17

No.	Type	Materials	ΔEMI SE (dB)	ΔEMI SE/t (dB/mm)	References
21	Shape change (stretching)	PEDOT:PSS/waterborne PU	4.00	26.67	S18
22	Temperature change	VO_2/CNF	34.10	17.95	S19
23	Temperature change	VO_2/EPM composites foam	3.00	0.86	S20
24	Temperature change	VO_2/PVDF-HFP	44.80	24.89	S21
25	Temperature change	$Ti_3C_2T_x$-WVO_2	13.90	6.95	S22
26	Temperature change	RGO/VO_2-300°C	7.00	2.48	S23
27	Temperature change	RGO/VO_2	46.00	7.93	S23
28	Temperature change	Graphene/PDMS	9.50	1.90	S24
29	Humidity change	Core-shell structural PNIPAM@p-PDA biomicrospheres	61.00	508.33	S25
30	Humidity change	Pyrolytic graphite-wet RGO/CNTs/PP non-woven spacer-pyrolytic graphite	22.00	11.00	S26
31	Electrochemical potential change	MXene film ($Ti_3C_2T_x$ electrode in 1 M H_2SO_4)/PET	2.20	21.57	S27
32	Electrochemical potential change	MXene film (V_2CT_x electrode in 1 M H_2SO_4)/PET	6.00	59.64	S27

No.	Type	Materials	ΔEMI SE (dB)	ΔEMI SE/t (dB/mm)	References
33	Rotation angle change	MXene/CNF aerogels	26.00	13.00	S28
34	Rotation angle change	MXene@wood	30.20	15.10	S29
35	Rotation angle change	Polyvinyl butyral/Ni-graphene/short-cut CF composite films	11.00	55.00	S30
36	Rotation angle change	CF reinforced polymer	39.00	70.91	S31
37	Rotation angle change	CF hybrid fabrics	4.50	4.37	S32
38	Rotation angle change	RGO@directional porous carbon	9.05	6.03	S33
39	Rotation angle change	CNT/NFC	38.50	4.81	S34
40	Rotation angle change	Co based amorphous wires	24.10	189.76	S35
41	Rotation angle change	MXene@PDA	17.00	566.67	S36
42	Rotation angle change	Balsa tangential-section carbonized wood	50.00	15.15	S37
43	Rotation angle change	Ordered RGO fiber film	26.00	866.67	S38
44	Rotation angle change	CNT-based spacer fabric with CVD	44.00	88.00	S39

No.	Type	Materials	ΔEMI SE (dB)	ΔEMI SE/t (dB/mm)	References
45	Rotation angle change	GAFM	26.54	1061.60	This work
46	Rotation angle change	GAFM/CF	54.33	196.85	This work

Note: nanoparticles (NP), trans-1,4-polyisoprene (TPI), ethylene–vinyl acetate copolymer (EVA), polypyrrole (PPy), silver (Ag), melamine foam (MF), nanowire (NW), poly(ethylene glycol) (PEG), wheat flour (WF), carbon nanotube (CNT), polyurethane (PU), poly-dopamine (PDA), polydimethylsiloxane (PDMS), reduced graphene oxide (RGO), polyacrylamide (PAM), 3 dimension (3D), styrene-(ethylenebutylene)-styrene (SEBS), thermoplastic polyurethane (TPU), cellulose nanofibrils (CNF), poly(3,4-ethylenedioxythiophene)/poly(styrenesulfonate) (PEDOT:PSS), carbon fiber (CF), vanadium dioxide (VO_2), expanded polymer microsphere (EPM), poly (vinylidenefluoride-co-hexafluoropropylene (PVDF-HFP), poly(N-isopropylacrylamide)@ porous polydopamine (PNIPAM@p-PDA), polyprolene (PP), poly(ethylene terephthalate) (PET), nano-brillated cellulose (NFC), chemical vapor deposition (CVD).

The thickness of the sample includes the thickness of the substrate.

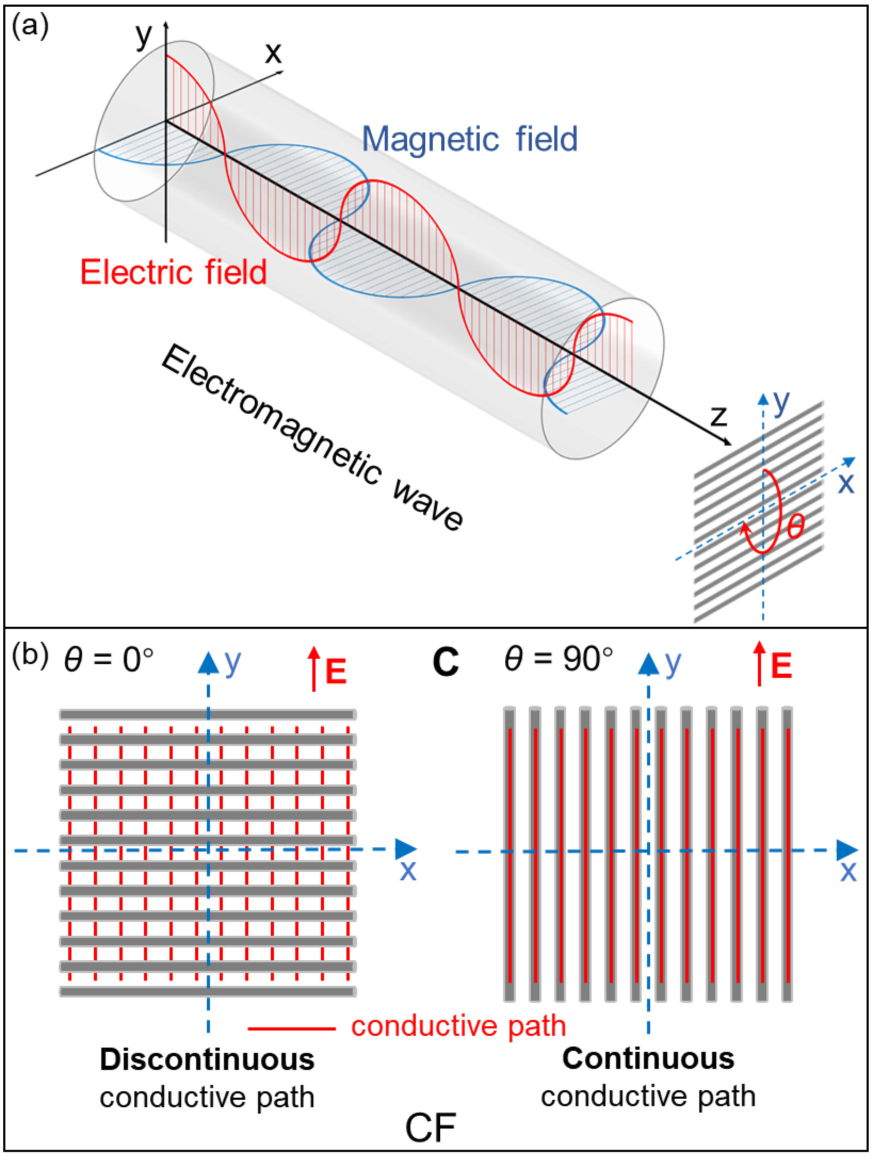

Supplementary Fig. 16. Schematic diagram of the EMI SE manipulation mechanism of CF after the angle between the CF fibers' axis direction and electromagnetic (EM) waves changes. (a) Schematic diagram illustrating the electric and magnetic fields of incident electromagnetic (EM) waves and the angle between the fiber axis direction of CF and the direction of the electric field. (b) Discontinuous conductive path of CF when $\theta = 0°$. (c) Continuous conductive path of CF when $\theta = 90°$.

The maneuverable EMI shielding effectiveness (EMI SE) of CF originates from its

anisotropic structure. According to Maxwell's equations, incident electromagnetic (EM) waves induce surface current density on the conductor's surface, thereby generating a perpendicular magnetic field due to the time-varying nature of the EM waves (see **Supplementary Fig. 16**). Additionally, a secondary electric field emerges in opposition to the incident EM wave's electric field, leading to attenuation of the incident electromagnetic energy. Aligning the direction of CF parallel to the incident EM wave's electric field maximizes the continuity of the conductive path, enhancing the induced surface current density on CF's surface and thus improving EM wave attenuation efficiency (see **Supplementary Fig. 16**). Conversely, when CF is perpendicular to the electric field direction, discontinuities in the conductive path significantly reduce the induced current density, resulting in weaker attenuation of EM waves.

Supplementary Note 1. Experimental details

S1.1 Characterizations of samples

X-ray diffraction (XRD) patterns of the graphene assembled film (GAF) samples were obtained using an X-ray diffractometer (D/MAX-RB RU-200B) equipped with Cu-$K\alpha$ radiation (wavelength λ = 0.154178 nm). The microstructure of graphene oxide (GO) sheets was characterized using atomic force microscopy (AFM) performed on an instrument from Asylum Research (Oxford Instruments, Cypher ES). High-resolution transmission electron microscopy (HRTEM, JEM-2100F) was employed to investigate the morphology of the prepared samples. Surface morphology and structural features were examined using field emission scanning electron microscopy (FESEM, JSM-7100F). Raman spectroscopy was conducted for the analysis of Raman shifts utilizing a Raman microscope (RENISHAW). Small-angle X-ray scattering (SAXS) images of the GAF were acquired using the Xeuss 3.0 SAXS/WAXS System with incident Cu-$K\alpha$ X-ray radiation (8.05 keV) under vacuum conditions (<1 mbar). The detector employed was the Eiger2R 1M model, with a pixel edge length of 75 μm.

S1.2 Electrical conductivity measurements of samples

The electrical conductivity of the GAF is measured by a Four-Point Probes Resistivity Measurement System (Probes Technology, RTS-9). The specific testing procedure is outlined as follows:

1. Surface Resistivity Test

Sample size: > 10 mm × 10 mm, probe spacing: S = 1 mm.

Firstly, the current (I) is applied from probe 1 to probe 4, while the voltage between probe

2 and 3 is denoted as V_{23+}.

Secondly, reversing the current direction, I is directed from probe 4 to probe 1, and the corresponding voltage between probe 2 and 3 is recorded as V_{23-}.

Thirdly, the mean value of the forward and reverse voltages is calculated as $V_{23} = (V_{23+} + V_{23-})/2$.

Fourthly, surface resistance ($R_□$) is determined using the formula: $R_□ = K \times (V_{23} / I)$, where K represents the correction factor.

2. Electrical Conductivity Test

Firstly, the volume resistivity (ρ) is calculated as: $\rho = R_□ \times W \times F(W/S)/10$ (Ω·cm), where W denotes the sample thickness, and $F(W/S)$ represents the thickness correction factor.

Secondly, the electrical conductivity (σ) is subsequently determined as: $\sigma = 1/\rho$.

S1.3 Electromagnetic interference shielding effectiveness measurements of samples

We performed EMI SE measurements using the radiated field method,[S40-S41] placing the GAFM in the far-field region between two horn antennas (details see **Supplementary Note 3. The EMI SE measurement procedures**). The EMI characteristics were evaluated by measuring the transmission coefficient between the two antennas, constrained by time gating. By changing the polarization direction of the horn antennas, we measured the response of the GAFM to electromagnetic waves with different polarizations.

Supplementary Note 2. The principle of high-polarization sensitivity of GAFM

The principle can be demonstrated by analyzing the electric field and surface distribution of the metamaterial. As an example, in the Y-polarization direction, the S-parameters exhibit two clear resonance points, as illustrated in **Supplementary Fig. 17.** Due to the Y-polarized incident electromagnetic wave, the electric potentials on the two long sides of the metamaterial are different. **Supplementary Fig. 18(a)** illustrates that the electric field on the metamaterial at 10 GHz induced by the Y-polarized wave exhibits a symmetric distribution with respect to the Y-axis. In the condition where the structure length equals half the wavelength, the electric field antinodes coincide. According to the surface current distribution shown in the **Supplementary Fig. 18(b)**, when the current path is an odd multiple of half the wavelength, the antinodes of the current wave coincide, leading to maximum current amplitude. At this point, both the voltage and current amplitudes reach their maximum values, indicating that the system is in a resonant state. Therefore, it is shown that the structure does produce 1/2 wavelength resonance under the excitation of Y-polarized wave.

If the length of the structure corresponds to the quarter wavelength, this spatial wave incidence is different from the resonance of the traveling wave structure (such as the 1/4 resonator of the microstrip). The electric field and current amplitudes cannot achieve antinode superposition, as illustrated in **Supplementary Fig. 19(a)** and **Supplementary Fig. 19(b)**. As a result, resonance cannot occur, making it impossible to achieve frequency-selective behavior. In other words, the resonant frequencies of our metamaterial occur at odd multiples of half wavelength, while no resonance is observed at the quarter-wavelength condition (5 GHz), which can be demonstrated by reflection coefficient shown in **Supplementary Fig. 17(a)**.

To further verify the validity of our design principle, we simulated the electric field and current distributions of the metamaterial at a higher-order resonant frequency (25 GHz), as illustrated in **Supplementary Fig. 20(a)** and **20(b)**. At this frequency, the length of the metamaterial corresponds to 1.5 wavelengths (odd multiples of half wavelength), which is consistent with our design concept.

To demonstrate the polarization sensitivity of the designed metamaterial, we simulated the resonant frequencies in different polarization directions, as shown in **Supplementary Fig. 21(a)**. For the X-polarization direction, the resonance still occurs when the length of the short side of the metamaterial corresponds to half wavelength, and the antinodes are superimposed as displayed in **Supplementary Fig. 21(b)**. There was no quarter-wavelength resonance at low frequencies either.

The difference in geometric dimensions along the X and Y axes imparts polarization sensitivity to the metamaterial, resulting in clearly distinguishable resonant frequencies in different polarization across a frequency range.

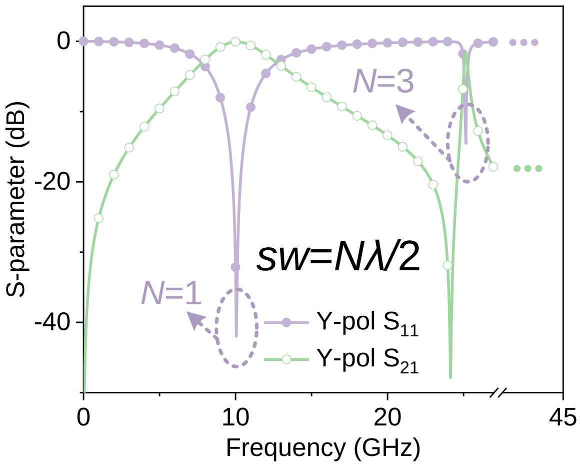

Supplementary Fig. 17. Simulated S-parameters (S_{11} and S_{21}) in Y-polarization direction.

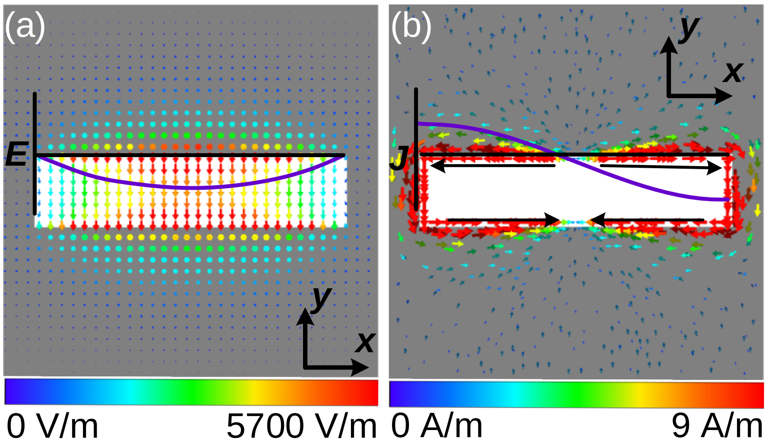

Supplementary Fig. 18. (a) Electric field distribution and (b) surface current distribution at 10 GHz.

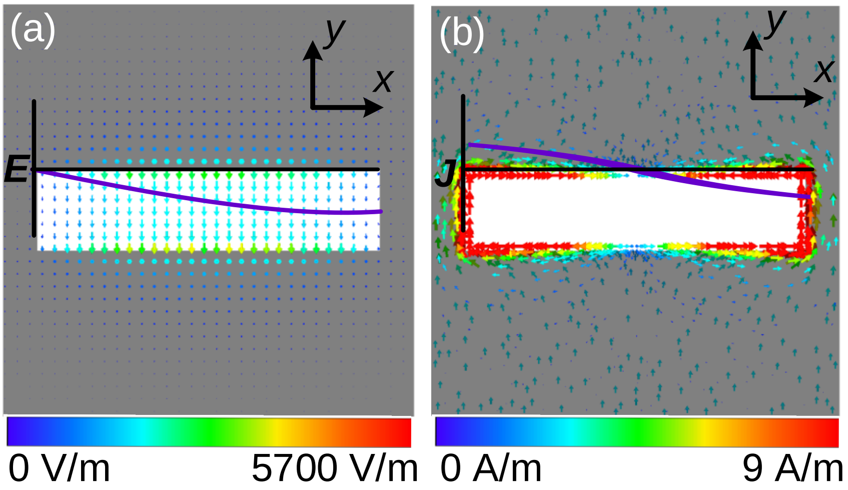

Supplementary Fig. 19. (a) Electric field distribution and (b) surface current distribution at 5 GHz.

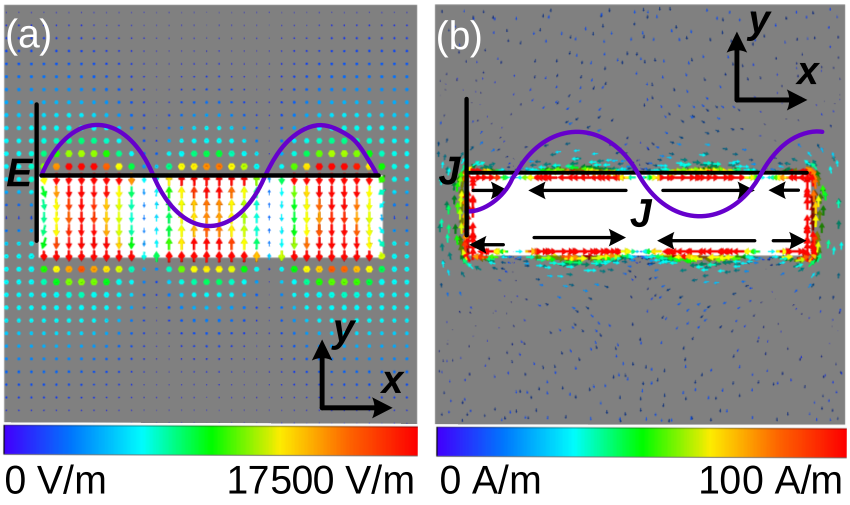

Supplementary Fig. 20. (a) Electric field distribution and (b) surface current distribution at 25 GHz.

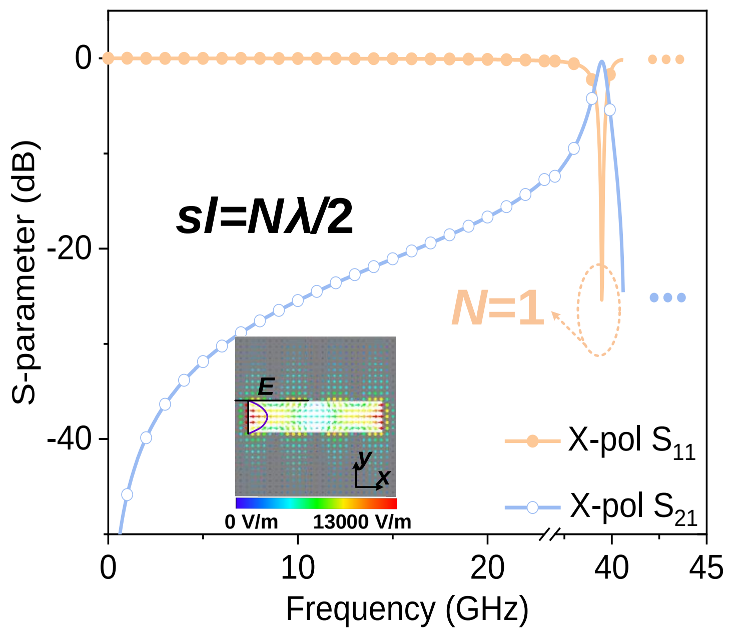

Supplementary Fig. 21. Simulated S-parameters in X-polarization direction. The inset is surface current distribution at 40 GHz.

Supplementary Note 3. The EMI SE measurement procedures

Electromagnetic interference (EMI) shielding effectiveness (SE) is measured using the radiated field method, which is a standard technique commonly employed for metasurface reflection or transmission characterization. Taking GAFM as an example, the specific steps of its EMI SE measurement are as follows:

1 Set up the test environment

In the radiated field method, the transmission characteristics of the sample are measured by placing it between the transmitting and receiving antennas. The experimental setups for EMI SE measurement of the proposed GAFM are illustrated in **Supplementary Fig. 22**. To ensure that the plane wave is normally incident on the metamaterial, the measurement must satisfy the far-field condition—specifically, the sample under measurement should be located within the far-field region of both the transmitting and receiving antennas. The far-field calculation conditions are：

$$R \geq \frac{2D^2}{\lambda} \tag{1}$$

Where R is the minimum distance between the antenna and the sample, D is the diameter of the antenna aperture, and λ is the operating wavelength. The distance between the reflector and the two horn antennas is 0.3 m. A $216\,\mathrm{mm} \times 216\,\mathrm{mm}$ rectangular hole is made in the absorber, into which the sample is inserted for EMI SE measurement. This configuration helps reduce diffraction propagation. To ensure the accuracy of the experiment, the entire measurement in this work was carried out in the anechoic chamber.

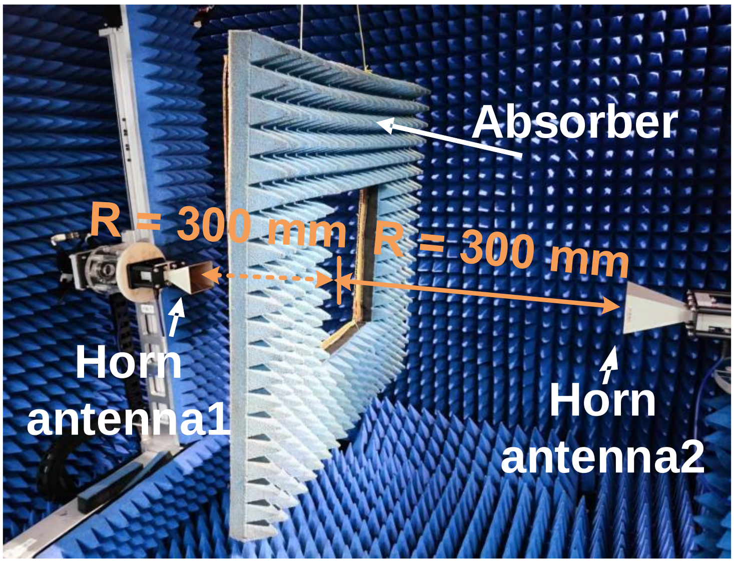

Supplementary Fig. 22. Experimental setup for EMI SE measurement calibration.

2 Calibrate the system and apply time gating

The transmission coefficients through air were measured without a sample in place as a reference baseline. In addition, time gating is applied by converting the frequency-domain S-parameters into the time domain via a Fast Fourier Transform (FFT). In the time domain, the signal directly associated with the sample along the main propagation path (the first transmission wave) can be identified and isolated. Stray signals in other delay intervals are effectively suppressed. The isolated signal is then transformed back into the frequency domain using an Inverse Fourier Transform (IFT) to obtain purified transmission coefficient data. This process eliminates the influence of stray reflections, diffraction, and other disturbances on the measurement results. The **Supplementary Fig. 23** illustrates the configuration of the time gating setup. We use the transmission coefficient obtained after applying time gating, as the reference (full transmission) for subsequent measurements.

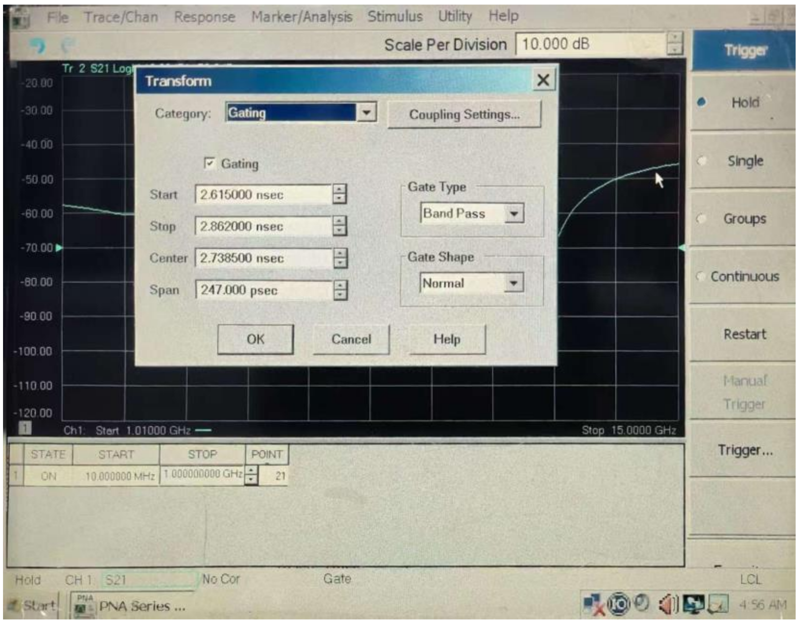

Supplementary Fig. 23. Configuration of the time gating window.

3 Obtain transmission coefficient

Place the metamaterial in the far field between the two horn antennas, with its shorter side aligned parallel to the polarization direction of the horns, as displayed in **Supplementary Fig. 24**. The EM wave is transmitted by horn antenna 1 and passed through the sample to be received by horn antenna 2. Through this process, the transmission coefficient of the sample is obtained.

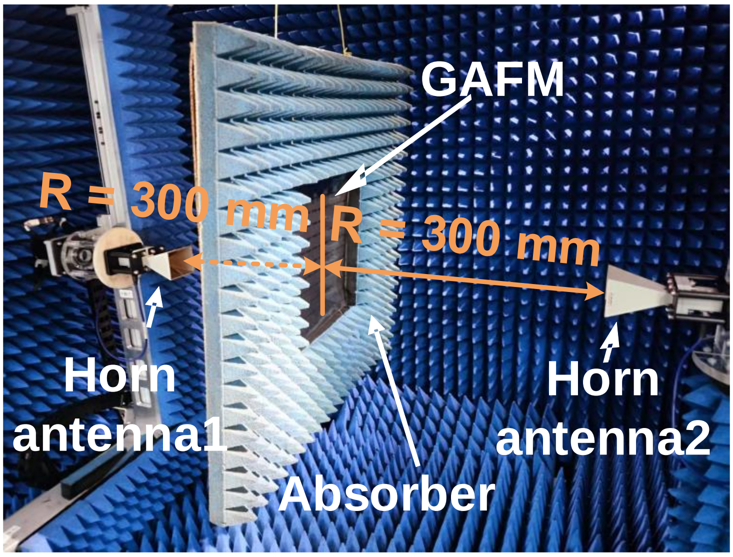

Supplementary Fig. 24. Experimental setup for testing GAFM in Y-polarization direction.

4 Rotate different angles and measure EMI characteristics

As shown in **Supplementary Fig. 25**, we rotate the two horn antennas to different angles and measure the EMI characteristics following the same steps. The measured original transmission coefficient (S_{21}) and the corresponding EMI SE result distribution are shown in **Supplementary Fig. 26(a)** and **26(b)**. The S_{21} parameter reflects the signal attenuation through the structure. A smaller S_{21} value indicates diminished signal penetration, corresponding to enhanced EMI SE.

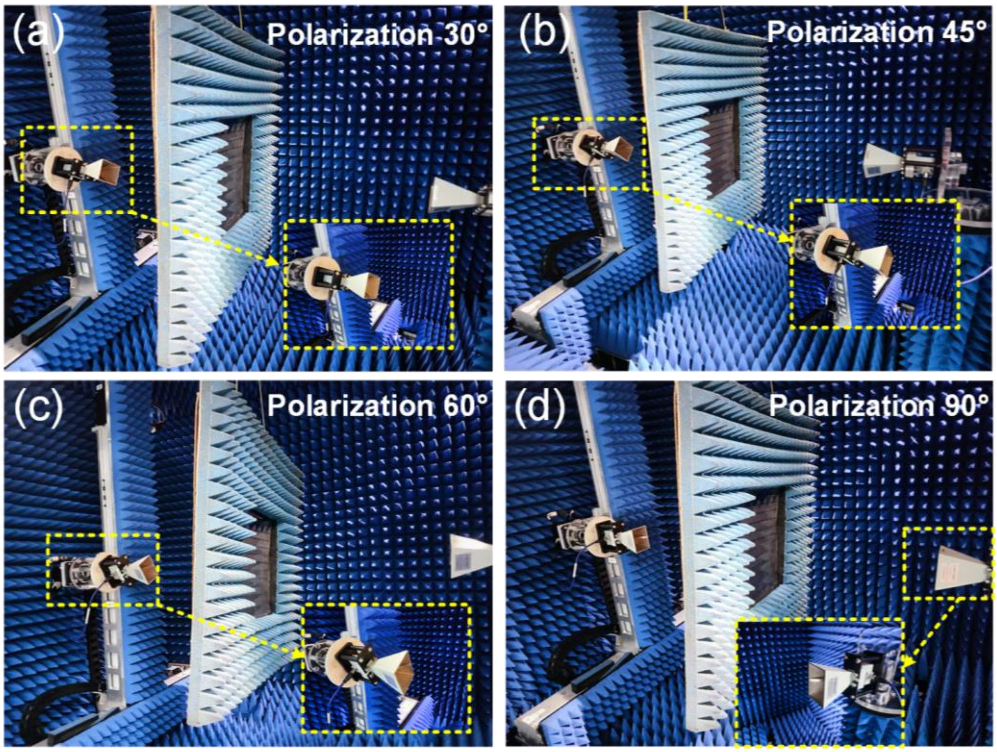

Supplementary Fig. 25. Experimental setup for metamaterial measurements under various polarization angles: (a) 30 °, (b) 45 °, (c) 60 °, and (d) 90 ° polarization.

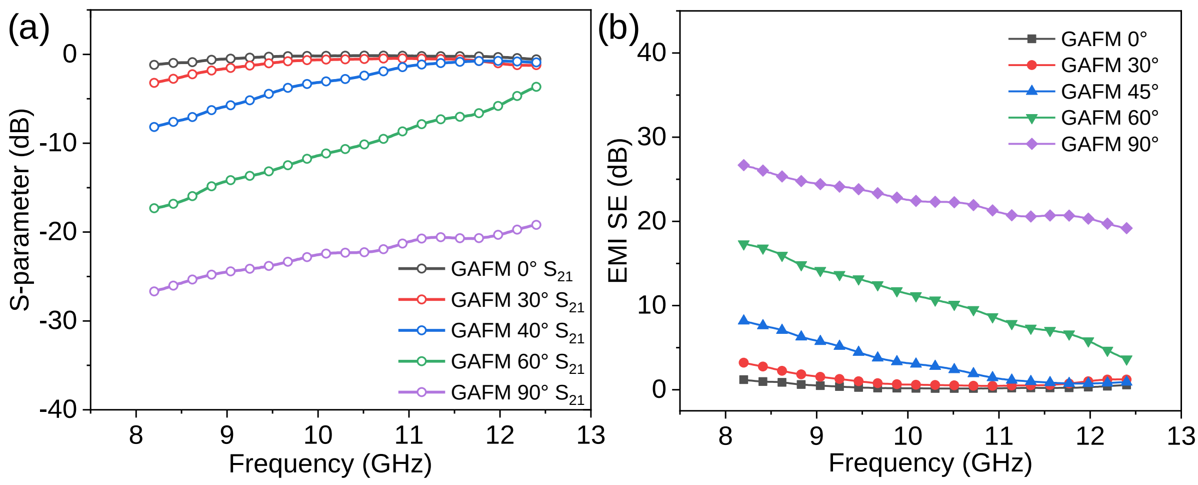

Supplementary Fig. 26. (a) The measured transmission coefficient (S_{21}). (b) The corresponding EMI SE result.

Supplementary References

[S1] L Liu, X. *et al.* Off/on switchable smart electromagnetic interference shielding aerogel. *Matter* **4**, 1735–1747 (2021).

[S2] Jia, X., Shen, B., Zhang, L. & Zheng, W. Construction of shape-memory carbon foam composites for adjustable EMI shielding under self-fixable mechanical deformation. *Chem. Eng. J.* **405**, 126927 (2021).

[S3] Chen, J. *et al.* Multifunctional shape memory foam composites integrated with tunable electromagnetic interference shielding and sensing. *Chem. Eng. J.* **466**, 143373 (2023).

[S4] He, Y. *et al.* Multifunctional phase change composites based on elastic MXene/silver nanowire sponges for excellent thermal/solar/electric energy storage, shape memory, and adjustable electromagnetic interference shielding functions. *ACS Appl. Mater. Interfaces* **14**, 6057–6070 (2022).

[S5] Chen, Y., Liu, Y., Li, Y. & Qi, H. Highly sensitive, flexible, stable, and hydrophobic biofoam based on wheat flour for multifunctional sensor and adjustable EMI shielding applications. *ACS Appl. Mater. Interfaces* **13**, 30020–30029 (2021).

[S6] Wang, G. *et al.* Structural design of compressible shape-memory foams for smart self-fixable electromagnetic shielding with reduced reflection. *Mater. Today Phys.* **22**, 100612 (2022).

[S7] Wang, S., Wang, Z., Zheng, S. Y. & Yang, J. Multifunctional heterostructured composite foam with tunable electromagnetic interference shielding. *Compos. Sci. Technol.* **248**, 110482 (2024).

[S8] Li, J. *et al.* Oriented magnetic liquid metal-filled interlocked bilayer films as

multifunctional smart electromagnetic devices. *Nano Res.* **16**, 1764–1772 (2023).

[S9] Shen, B., Li, Y., Zhai, W. & Zheng, W. Compressible graphene-coated polymer foams with ultralow density for adjustable electromagnetic interference (EMI) shielding. *ACS Appl. Mater. Interfaces* **8**, 8050–8057 (2016).

[S10] Bian, J. *et al.* High-strain-sensitive dynamically adjustable electromagnetic interference shielding elastomer with pre-linked nickel chains. *Sci. China Mater.* **67**, 629–641 (2024).

[S11] Guo, H. *et al.* Tough, stretchable dual-network liquid metal-based hydrogel toward high-performance intelligent on-off electromagnetic interference shielding, human motion detection and self-powered application. *Nano Energy* **114**, 108678 (2023).

[S12] Zhu, R. *et al.* Anisotropic magnetic liquid metal film for wearable wireless electromagnetic sensing and smart electromagnetic interference shielding. *Nano Energy* **92**, 106700 (2022).

[S13] Yao, B. *et al.* Highly stretchable polymer composite with strain-enhanced electromagnetic interference shielding effectiveness. *Adv. Mater.* **32**, 1907499 (2020).

[S14] Yu, D. *et al.* A super-stretchable liquid metal foamed elastomer for tunable control of electromagnetic waves and thermal transport. *Adv. Sci.* **7**, 2000177 (2020).

[S15] Liu, Z. *et al.* A general approach for buckled bulk composites by combined biaxial stretch and layer-by-layer deposition and their electrical and electromagnetic applications. *Adv. Electron. Mater.* **5**, 1800817 (2019).

[S16] Feng, D., Xu, D., Wang, Q. & Liu, P. Highly stretchable electromagnetic interference (EMI) shielding segregated polyurethane/carbon nanotube composites fabricated by microwave selective sintering. *J. Mater. Chem. C* **7**, 7938–7946 (2019).

[S17] Zhang, M. *et al.* Stretchable liquid metal electromagnetic interference shielding coating materials with superior effectiveness. *J. Mater. Chem. C* **7**, 10331–10337 (2019).

[S18] Li, P., Du, D., Guo, L., Guo, Y. & Ouyang, J. Stretchable and conductive polymer films for high-performance electromagnetic interference shielding. *J. Mater. Chem. C* **4**, 6525–6532 (2016).

[S19] Liao, S.-Y. *et al.* Intelligent shielding material based on VO_2 with tunable near-field and far-field electromagnetic response. *Chem. Eng. J.* **464**, 142596 (2023).

[S20] Liao, S. *et al.* Reversible switching between microwave absorption and EMI shielding of VO_2 composite foam. *Small* **20**, 2402841 (2024).

[S21] Liang, S. *et al.* Tunable high-performance electromagnetic interference shielding of VO_2 nanowires-based composite. *ACS Appl. Mater. Interfaces* **16**, 21024–21033 (2024).

[S22] Qian, H. *et al.* Pushing electromagnetic interference shielding self-enhanced based on smart $Ti_3C_2T_x$-WVO_2 thermal management composite. *Carbon* **210**, 118081 (2023).

[S23] Cheng, Z. *et al.* Intelligent off/on switchable microwave absorption performance of reduced graphene oxide/VO_2 composite aerogel. *Adv. Funct. Mater.* **32**, 2205160 (2022).

[S24] Gao, W. *et al.* High-efficiency electromagnetic interference shielding realized in nacre-mimetic graphene/polymer composite with extremely low graphene loading. *Carbon* **157**, 570–577 (2020).

[S25] Li, C. *et al.* Succulent-inspired implicit structural change for smart “ON/OFF” switchable and flexible EMI shielding coating. *ACS Appl. Mater. Interfaces* **16**, 12939–12950 (2024).

[S26] Wang, Y. *et al.* Hydro-sensitive sandwich structures for self-tunable smart

electromagnetic shielding. *Chem. Eng. J.* **344**, 342–352 (2018).

[S27] Han, M. *et al.* Electrochemically modulated interaction of MXenes with microwaves. *Nat. Nanotechnol.* **18**, 373–379 (2023).

[S28] Zeng, Z. *et al.* Nanocellulose-MXene biomimetic aerogels with orientation-tunable electromagnetic interference shielding performance. *Adv. Sci.* **7**, 2000979 (2020).

[S29] Wei, Y. *et al.* Highly anisotropic MXene@wood composites for tunable electromagnetic interference shielding. *Compos. Part A Appl. Sci. Manuf.* **168**, 107476 (2023).

[S30] Wen, B., Wang, X. & Zhang, Y. Ultrathin and anisotropic polyvinyl butyral/Ni-graphite/short-cut carbon fibre film with high electromagnetic shielding performance. *Compos. Sci. Technol.* **169**, 127–134 (2019).

[S31] Hong, J. & Xu, P. Electromagnetic interference shielding anisotropy of unidirectional CFRP composites. *Materials.* **14**, 1907 (2021).

[S32] Hong, X. *et al.* Polarization selection characteristics of carbon fiber orientation and interweaving for electromagnetic interference shielding behaviors. *Text. Res. J.* **92**, 269–283 (2022).

[S33] Liu, Z. *et al.* Gradient in-plane oriented porous carbon inspired by fabrication of toasts for elegant EMI shielding performance. *Carbon* **207**, 136–143 (2023).

[S34] Zeng, Z. *et al.* Nanocellulose assisted preparation of ambient dried, large-scale and mechanically robust carbon nanotube foams for electromagnetic interference shielding. *J. Mater. Chem. A* **8**, 17969–17979 (2020).

[S35] Dai, X. *et al.* A smart amorphous wire composite with tunable electromagnetic shielding. *Small Struct.* **5**, 2300405 (2024).

[S36] Deng, Z. *et al.* Controllable surface-grafted MXene inks for electromagnetic wave modulation and infrared anti-counterfeiting applications. *ACS Nano* **16**, 16976–16986 (2022).

[S37] Dai, Z. *et al.* Highly anisotropic carbonized wood as electronic materials for electromagnetic interference shielding and thermal management. *Adv. Electron. Mater.* **9**, 2300162 (2023).

[S38] Xu, L. *et al.* Ultrathin, ultralight, and anisotropic ordered reduced graphene oxide fiber electromagnetic interference shielding membrane. *Adv. Mater. Technol.* **6**, 2100531 (2021).

[S39] Li, D. *et al.* 3D-structured carbon nanotube fibers as ultra-robust fabrics for adaptive electromagnetic shielding. *Nano Res.* **17**, 8521–8530 (2024).

[S40] Wang, J., Yang, R. Generating High-Purity Directive Circularly Polarized Beams From Conformal Anisotropic Holographic Metasurfaces. *IEEE T. Antenn. Propag.* **70**, 10718-10723 (2022).

[S41] International Electrotechnical Commission. Specification for radio disturbance and immunity measuring apparatus and methods – Part 2-3: Methods of measurement of disturbances and immunity – Radiated disturbance measurements. *IEC CISPR* **16-2-3**, Ed. 4.0 (2016).
